# Gifts in Kind: Their Valuation in Hospital Accounts

**Published:** 1916-11-18

**Authors:** 


### GIFTS IN KIND.

### Their Valuation in Hospital Accounts.

THE King's Fund regulation respecting the valuation for accountancy purposes of presents of food and other articles appeared for the first time in the issue of the *Red Book* (August 1914). The wording implies that every gift should be valued; but a circular dated June 1915, issued by the three Metropolitan Central Funds, explains that game, fruit, or articles not in general use for patients or nurses need not be valued for purposes of account; the rule being intended to provide for the valuation of the gifts of Linen Leagues, of food, and other presents relieving ordinary expenditure.

Last year was the first complete twelve months in which the effect of the new rule was shown in the accounts of hospitals in London. In examining a number of reports, it is found that the gross value of gifts received in 1915 at certain institutions varied in a surprising way. A few instances of the results are quoted:—

| Hospital | Number of Beds | Value of Gifts in Kind as Shown in Annual Report, 1915 |
|---|---|---|
| St. Thomas's ... ... ... | 983 | £1,171 |
| London ... ... ... | 922 | 1,212 |
| Guy's ... ... ... ... | 643 | 436 |
| Middlesex ... ... ... | 350 | 693 |
| St. George's ... ... ... | 349 | 2,188 |
| St. Mary's ... ... ... | 298 | 260 |
| Great Northern Central ... | 224 | 544 |
| Westminster ... ... ... | 213 | 352 |
| Royal Free ... ... ... | 175 | 84 |
| National Hospital for the Paralysed and Epileptic | 160 | 234 |

One hospital of considerable size shows no valuation in the accounts, but the report of the board of management mentions the receipt of goods worth £350. Another important hospital makes the credit under "Donations" instead of, as directed by the rule, under "Other Receipts."

A gift, within the meaning of the rule, it seems, might be defined as something given which saves something of equal value being bought; but this definition must be qualified, because such gifts as game must be excluded from a valuation although they undoubtedly save the consumption of other eatables. However, the definition is of use in determining the rate of valuation. What has the gift saved? Some food, linen, or other commodity which would have been bought on, or shortly after, the date of the gift. Therefore the rate of valuation should be the price paid at or about that date under running contracts, or, if there are no contracts, at market price.

But as the valuation appears as expenditure as well as income, the average cost of the occupied bed is affected; why should not a hospital having a disadvantageous contract value at the price of the day? This may be only, one instance of an accountency difficulty as to valuation; it suggests the possibility of important variations in method.

During the war, hospitals are receiving gifts in kind in considerable quantities from Colonial Governments, through Chambers of Commerce, from egg-collecting agencies and other sources. Especially at the present time is the accountancy question important, and if our accounts are to be presented in a form which admits of comparison, it is essential that there should be no haziness as to method, otherwise the basis upon which certain funds are allocated must be unreliable. The figures we have quoted, although, possibly approximately accurate, and the points raised in this article, seem to present a case for discussion as to whether the directions of the Metropolitan Funds are sufficiently explicit, or whether the methods of recording the receipt of these goods, and the way in which their value is dealt with in the books of the respective hospitals, are carried out under a uniform system.

